# Genome Sequence of *Acinetobacter baumannii* Strain D36, an Antibiotic-Resistant Isolate from Lineage 2 of Global Clone 1

**DOI:** 10.1128/genomeA.01478-15

**Published:** 2015-12-17

**Authors:** Mohammad Hamidian, Jane Hawkey, Kathryn E. Holt, Ruth M. Hall

**Affiliations:** a School of Molecular Bioscience, The University of Sydney, Sydney, Australia; b Department of Biochemistry and Molecular Biology and Centre for Systems Genomics, University of Melbourne, Melbourne, Australia; c Department of Biochemistry and Molecular Biology, Bio21 Molecular Science and Biotechnology Institute, University of Melbourne, Melbourne, Australia

## Abstract

Multiply antibiotic-resistant *Acinetobacter baumannii* isolate D36 was recovered in Australia in 2008 and belongs to a distinct lineage of global clone 1 (GC1). Here, we present the complete 4.13 Mbp genome sequence (chromosome plus 4 plasmids), generated via long read sequencing (PacBio).

## GENOME ANNOUNCEMENT

*Acinetobacter baumannii* isolate D36 was isolated from a soldier in 2008 at North Shore Private Hospital, Sydney, Australia (1). It is resistant to several antibiotics, imipenem and meropenem, ceftazidime and cefotaxime, fluoroquinolones, sulfonamides, and aminoglycosides gentamicin, kanamycin, tobramycin, and neomycin (2). It was previously shown to represent a distinct lineage of global clone 1 (GC1) having AbaR4 rather than an AbaR0 derivative in the *comM* gene (1).

DNA was subjected to sequencing on 2 PacBio single-molecule real-time (SMRT) cells (chemistry version C2-P4) at DNA Link (South Korea). A total of 113,387 reads were obtained with an average length of 7,984 bp and average quality 0.827. The PacBio reads were assembled *de novo* using SMRT Analysis Suite v2.3.0 and the HGAP.3 algorithm with default parameters.

The assembled contiguous chromosomal sequence was 4,063,596 bp and a segment of 51.7 kb between two copies of the rRNA genes on either side of the replication origin is inverted relative to other *A. baumannii* genomes. Plasmid contigs were circularized using PCR, producing four plasmids pD36-1 to pD36-4 of 4,754 bp, 6,078 bp, 9,276 bp, and 47,457 bp, respectively. Protein coding rRNA and tRNA genes were annotated using RAST (3), and the antibiotic resistance and polysaccharide biosynthesis loci, transposons, insertion sequences, phage genomes, and plasmids were annotated manually.

The genome sequence confirms that D36 is a member of global clone 1 (GC1), one of the resistant clones found on all inhabited continents. It belongs to CC1 (ST81) in the Institute Pasteur MLST scheme (4). In the Oxford scheme (5) it is ST498(ST247), a single locus variant of ST231(ST109) differing only in *gpi* which resides in the capsule locus, and D36 carries the KL12 capsule locus (6, 7) and the OCL1 outer core locus (8). A 49.9-kb transposon, designated Tn*6171,* carries a potential siderophore synthesis gene cluster previously seen only in ATCC 17978 (9). Tn*6171* encodes transposition proteins related to those of Tn*7.* The chromosome includes 18 copies of the insertion sequence (IS) ISAba1, three of ISAba14 and one each of ISAba12 and IS*26.* Three potential integrated phage genomes of 95,928 bp, 36,399 bp, and 36, 438 bp were also identified using PHAST (10).

Carbapenem resistance is due to the presence of the *oxa23* gene in AbaR4 in *comM* (1) and third generation cephalosporin resistance is due to increased transcription of the *ampC* gene from an upstream ISAba1 (11). The 6,078-bp plasmid pD36-2 is pRAY* carrying the *aadB* gentamicin, kanamycin, and tobramycin resistance gene cassette (2). Plasmid pD36-4 carries the *sul2* sulfonamide resistance gene, the *aphA1a* kanamycin/neomycin resistance gene in Tn*4352,* a *mer* module conferring resistance to mercuric ions, and 11 IS. pD36-1 and pD36-3 are cryptic.

The genome sequence of D36 will underpin studies of the evolution of the second branch of the GC1 clonal complex.

### Nucleotide sequence accession numbers.

The complete genome sequence has been deposited in DDBJ/ENA/GenBank under the accession numbers CP012952 (chromosome) and CP012953 to CP012956 (plasmids). The versions described in this paper are the first versions, CP012952.1 to CP012956.1.
